# Effects of pharmacologic sclerostin inhibition or testosterone administration on soleus muscle atrophy in rodents after spinal cord injury

**DOI:** 10.1371/journal.pone.0194440

**Published:** 2018-03-26

**Authors:** Ean G. Phillips, Luke A. Beggs, Fan Ye, Christine F. Conover, Darren T. Beck, Dana M. Otzel, Payal Ghosh, Anna C. F. Bassit, Stephen E. Borst, Joshua F. Yarrow

**Affiliations:** 1 Research Service, Malcom Randall VA Medical Center, North Florida/South Georgia Veterans Health System, Gainesville, FL, United States of America; 2 School of Kinesiology, Auburn University, Auburn, AL, United States of America; 3 Department of Cell Biology and Physiology, Edward Via College of Osteopathic Medicine-Auburn Campus, Auburn, AL, United States of America; 4 Brain Rehabilitation Research Center, Malcom Randall VA Medical Center, North Florida/South Georgia Veterans Health System, Gainesville, FL, United States of America; 5 Department of Nutrition, Food and Exercise Sciences, Florida State University, Tallahassee, FL, United States of America; 6 Orthopedics Department, Shriners Hospital for Children, Montreal, QC, Canada; 7 Department of Applied Physiology and Kinesiology, University of Florida, Gainesville, FL, United States of America; 8 Division of Endocrinology, Diabetes and Metabolism, University of Florida College of Medicine, Gainesville, FL, United States of America; University of Sydney, AUSTRALIA

## Abstract

Sclerostin is a circulating osteocyte-derived glycoprotein that negatively regulates Wnt-signaling after binding the LRP5/LRP6 co-receptors. Pharmacologic sclerostin inhibition produces bone anabolic effects after spinal cord injury (SCI), however, the effects of sclerostin-antibody (Scl-Ab) on muscle morphology remain unknown. In comparison, androgen administration produces bone antiresorptive effects after SCI and some, but not all, studies have reported that testosterone treatment ameliorates skeletal muscle atrophy in this context. Our purposes were to determine whether Scl-Ab prevents hindlimb muscle loss after SCI and compare the effects of Scl-Ab to testosterone enanthate (TE), an agent with known myotrophic effects. Male Sprague-Dawley rats aged 5 months received: (A) SHAM surgery (T_8_ laminectomy), (B) moderate-severe contusion SCI, (C) SCI+TE (7.0 mg/wk, im), or (D) SCI+Scl-Ab (25 mg/kg, twice weekly, sc). Twenty-one days post-injury, SCI animals exhibited a 31% lower soleus mass in comparison to SHAM, accompanied by >50% lower soleus muscle fiber cross-sectional area (fCSA) (p<0.01 for all fiber types). Scl-Ab did not prevent soleus atrophy, consistent with the relatively low circulating sclerostin concentrations and with the 91–99% lower LRP5/LRP6 gene expressions in soleus versus tibia (p<0.001), a tissue with known anabolic responsiveness to Scl-Ab. In comparison, TE partially prevented soleus atrophy and increased levator ani/bulbocavernosus (LABC) mass by 30–40% (p<0.001 vs all groups). The differing myotrophic responsiveness coincided with a 3-fold higher androgen receptor gene expression in LABC versus soleus (p<0.01). This study provides the first direct evidence that Scl-Ab does not prevent soleus muscle atrophy in rodents after SCI and suggests that variable myotrophic responses in rodent muscles after androgen administration are influenced by androgen receptor expression.

## Introduction

The musculoskeletal decline resulting from spinal cord injury (SCI) is precipitated by the neurologic insult and reduced loading in the paralyzed limbs [1]. However, the molecular signals that regulate muscle and bone loss after SCI require further elucidation. In our companion paper, we reported that stimulation of either the Wnt/β-catenin signaling pathway, via a monoclonal anti-sclerostin antibody (Scl-Ab), or the androgen signaling pathway, via testosterone-enanthate (TE), resulted in significant cancellous bone preservation in a rodent moderate-severe contusion SCI model, albeit via differing bone anabolic and antiresorptive mechanisms, respectively [2]. These results suggest that Wnt/β-catenin signaling and androgen signaling represent potential pathways influencing SCI-induced bone loss. Herein, we report the effects of these agents on sublesional skeletal muscles that were obtained from the animals examined in our companion paper because the Wnt/β-catenin signaling pathway [3] and the androgen signaling pathway produce anabolic effects in muscle [4], at least in the non-neurologically-impaired state, and because there is increasing recognition of biochemical bone-to-muscle crosstalk, as a mechanism through which musculoskeletal tissue is co-regulated [5,6]. In addition, the evaluation of off-target tissue responses remains important in the context of determining the systemic safety and/or efficacy of preclinical pharmacologic agents.

Sclerostin is an osteocyte-derived glycoprotein that is increased after SCI [7,8] and that acts as a negative regulator of bone formation. Specifically, sclerostin binds the low density lipoprotein receptor related protein complex (LRP5/LRP6), which inhibits both the canonical and non-canonical Wnt anabolic signaling pathways [3]. Sclerostin influences SCI-induced bone loss, as evidenced by (1) increased sclerostin mRNA expression in bone acutely after SCI [7], (2) mice with sclerostin gene deletion that do not exhibit bone loss after spinal cord transection [9], and (3) the ability pharmacologic sclerostin-inhibition to completely prevent cancellous bone loss in rats following SCI [2,10]. Others have suggested that sclerostin may also influence skeletal muscle [5], a supposition that is strengthened by the understanding that sclerostin is present in the circulation [8], that LRP5/LRP6 are expressed in human muscle [11,12], and that the Wnt/β-catenin signaling pathway is anabolic in muscle [3]. Interestingly, Huang et al recently reported that Wnt3a, an osteocyte-derived Wnt-signaling agonist, promoted C2C12 cell differentiation *in vitro* and that sclerostin co-incubation (100 ng/ml) prevented this effect [13], demonstrating that sclerostin negatively regulates Wnt-signaling in a mouse skeletal muscle cell line, at least when present in relatively high concentrations. The findings mentioned above and the observation that high circulating sclerostin occurs in humans acutely after SCI [8], suggests that sclerostin may influence muscle loss in this condition. However, we are unaware of any study that has evaluated LRP5/LRP6 expression in rodent muscle or whether Scl-Ab alters muscle morphology *in vivo*.

Testosterone also influences musculoskeletal integrity after SCI, as evidenced by (1) the high prevalence of low testosterone in men [14] and rodents after SCI [2,15,16], (2) the ability of androgens to prevent SCI-induced bone loss in young [16,17] and skeletally-mature animals [2,15], and (3) the ability of testosterone to improve lean mass [18,19] and muscle cross-sectional area in men with motor-complete SCI [20]. However, studies evaluating the myotrophic effects of androgens in rodent muscle after SCI have produced conflicting results, with some reporting that testosterone partially preserves hindlimb muscle mass [16,21,22] and others reporting no prevention of muscle atrophy [23,24]. Indeed, we have observed that testosterone-enanthate (TE) completely preserved mass of the sublesional non-weight bearing levator ani/bulbocavernosus (LABC) muscle complex after SCI [15], while producing a much less robust effect in the soleus [16]. These inconsistent myotrophic responses may be influenced by the differing SCI models (i.e., contusion vs transection), which allow for varying degrees of voluntary locomotor recovery, or by the specific muscle groups evaluated, which may exhibit differing androgen sensitivity due to their relative androgen receptor (AR) expressions.

The primary purposes of this study were to determine whether pharmacologic sclerostin inhibition can attenuate muscle atrophy occurring in a rodent moderate-severe contusion SCI model, and to assess relative expressions of LRP5 and LRP6 in the LABC, soleus, and tibia of rodents. A secondary purpose was to compare AR expression in LABC and soleus, as a means of explaining the differing myotrophic responses in rodent muscles after TE administration. We hypothesized that Scl-Ab would partially prevent SCI-induced muscle loss and that AR expression and the myotrophic response to TE would be more pronounced in LABC versus soleus.

## Material and methods

### Experimental design

We assessed sublesional weight bearing and non-weight bearing muscles from a companion experiment that evaluated the effects of Scl-Ab and TE on bone loss after SCI [2]. As described previously [2], 45 rats were blocked by initial weight and randomized into the following groups: (A) sham surgery (T_8_ laminectomy) (SHAM), (B) moderate-severe (250 kilodyne) contusion SCI (SCI), (C) SCI+Scl-Ab, or (D) SCI+TE. SCI+Scl-Ab animals received Scl-Ab (25 mg/kg twice weekly for 3 weeks, sc) while all other groups received saline vehicle (sc). SCI+TE animals received TE (7.0 mg/wk for 3 weeks, im), whereas all other groups received sesame oil vehicle (im). TE/sesame oil was administered under brief isoflurane anesthesia to ensure identical anesthesia exposure among groups, while no anesthesia was necessary for Scl-Ab/saline (sc) treatments. Animals were assessed for open-field locomotion by two blinded observers using the Basso-Beattie-Bresnahan (BBB) locomotor rating scale at weekly intervals. Animals were euthanized 21 days after surgery via an overdose of pentobarbital (120 mg/kg, ip), serum was collected by cardiac stick, and tissues were excised and weighed. The right soleus muscles were pinned at resting length on cork board, coated with Tissue-Tek® O.C.T. compound (Sakura Finetek, Torrance, CA), frozen on a slurry of liquid nitrogen-cooled 2-methylbutane, and stored at -80° C. Other tissues were snap frozen in liquid N and stored at -80° C.

### Animal care

Barrier-raised and specific pathogen-free male Sprague-Dawley rats aged 5 months were obtained from Charles River Laboratories (Wilmington, MA). Animals were individually housed in a temperature- and light-controlled room on a 12-hour light/dark cycle. Rats were fed rodent chow containing 3.1 kcal/g, distributed as 58% carbohydrate, 24% protein, and 18% fat (2018 Teklad Global 18% Protein Rodent Diet, Harlan Laboratories Inc., Indianapolis, IN) and tap water *ad libitum*. All experimental procedures conformed to the ILAR Guide to the Care and Use of Experimental Animals and were approved by the Institutional Animal Care and Use Committee at the Malcom Randall VA Medical Center.

### Surgery and postoperative care

As described previously in detail, the spinal cord was exposed by laminectomy under sterile conditions and a 250 kilodyne force was applied to the T_8_ segment of the spinal cord using the Infinite Horizons (IH) Impactor (Precision Systems and Instrumentation, Lexington, KY) to induce a moderate-severe mid-thoracic contusion SCI [2,15,16]. Post-operatively, rats received buprenorphine (0.05 mg/kg, sc) and ketoprofen (5.0 mg/kg, sc) to reduce pain and inflammation for 48 hours, and ampicillin was administered for 5 days after surgery. Postoperative care included assessments for signs of distress, weight loss, dehydration, fecal clearance, bladder dysfunction, and skin lesions. Bladders were expressed manually (2–3 times daily) until spontaneous voiding returned, which usually occurred within 2–3 weeks of surgery. Ringer's solution was provided sc to promote rehydration. A nutritional supplement (Jell-O cube with added protein and fat) and apples were provided to expedite body mass recovery.

### Animal model and study duration rationale

We have previously reported that moderate-severe contusion SCI produces extensive hindlimb muscle loss in young rats [16], with the majority of muscle atrophy occurring within 7 days of injury [25]. For this reason, we initiated pharmacologic therapy immediately post-surgery. Herein, we utilized 5-month-old skeletally-mature rats because most SCIs occur in adult men via a rapid contusion impact to the spinal cord.

### Drug selection rationale

We utilized a sclerostin-neutralizing monoclonal antibody, Scl-AbIII, from Amgen, Inc. (Thousand Oaks, CA, USA) and UCB (Brussels, Belgium), because circulating sclerostin is elevated in the SCI population acutely after injury [8], because sclerostin inhibits Wnt3a signaling in C2C12 skeletal muscle cells *in vitro* [13], because sclerostin mRNA is increased in bone in rodents after SCI [7], and because Scl-Ab is known to produce bone anabolic effects in rodent SCI models at the dose administered (25 mg/kg twice weekly for three weeks, sc) [2,10]. Testosterone-enanthate (Savient Pharmaceuticals, East Brunswick, NJ) was selected because a large proportion of men exhibit low testosterone after SCI [14], an effect that is also present in our rodent moderate-severe contusion SCI model [2,15,16], and because TE is known to prevent cancellous bone loss in young [16] and skeletally-mature rats after SCI [2], at the dose provided (7.0 mg/week, im), and to ameliorate hindlimb bone [26] and muscle loss after orchiectomy [27,28]. In addition, the TE dose and administration route are similar to a clinical trial we conducted in older hypogonadal men which reported increased fat-free mass, muscle strength, and bone mineral density in response to TE [29], demonstrating the clinical relevance of this treatment.

### Muscle histology

Serial (10 μm) transverse sections of the soleus mid-belly region were taken on a CM1850 cryostat (Leica Biosystems, Buffalo Grove, IL) and mounted on gelatin-coated glass slides. Immunohistochemistry (n = 6-8/group) was performed for determination of fiber cross-sectional area (fCSA) and fiber type distribution, as previously reported [25,30,31]. Antibodies used were as follows: rabbit anti-laminin (1:200; Thermo Scientific, Waltham, MA), rhodamine-conjugated goat anti-rabbit IgG (1:500), Alexa Fluor® 488 goat anti-mouse IgG (1:200), Alexa Fluor® 488 goat anti-mouse IgM (1:200) all from Life Technologies (Carlsbad, CA); and anti-MHC I (BA.D5), anti-MHC IIA (SC.71), anti-MHC IIB (BF.F3), and anti-MHC IIX (BF.35) obtained from the Developmental Studies Hybridoma Bank (Iowa City, IA). Image acquisition was performed on an Eclipse TE2000-S microscope (Nikon Instruments, Melville, NY) with the fiber type distribution and average fCSA determined, in a blinded manner by consensus of two investigators, from an analysis of 300–350 total fibers per animals with Image J (NIH).

### Gene expression

All instruments were treated with RNAse Zap prior to use and between samples. Tibiae from SHAM animals (n = 6) were cut into small pieces and pulverized with a liquid nitrogen cooled Spex Certiprep freezer mill (Edison, NJ). Soleus and LABC (n = 6) were pulverized via mortar and pestle in liquid nitrogen. All samples were immediately treated with Trizol reagent (Life Technologies, Carlsbad CA) and transported to the University of Florida Interdisciplinary Center for Biotechnology Research (ICBR) Core Laboratory, where total RNA was extracted and evaluated via real-time PCR analysis. The following commercially-available gene-specific primers were used (ThermoFisher Scientific, Carlsbad, CA): rat LRP5 (#Rn01451428_m1), rat LRP6 (#Rn01492711_m1), rat AR (#Rn00560747_m1), and rat GAPDH (#Rn01775763_m1).

### Statistical analysis

Results are reported as mean±SEM, with the threshold for significance defined as *p* < 0.05. One-way ANOVAs were used to determine if differences existed among groups for outcomes assessed at only one timepoint. Several outcomes reported in our companion paper [2] were also evaluated at multiple timepoints (i.e., body mass and BBB score) using mixed-model repeated measures ANOVAs. Tukey's *post hoc* tests were performed, when appropriate, to identify where differences existed. Bivariate analyses using Pearson’s correlation coefficients were used to determine linear associations between BBB score and muscle characteristics and between serum testosterone and muscle/prostate characteristics for specific variables of interest. The Holm-Bonferroni correction was utilized to correct for potential type I error that can occur when performing multiple comparisons. Data were analyzed with the SPSS version 24.0.0 statistical software package (IBM, Chicago, IL, USA).

## Results

### Injury severity and animal characteristics

Functional and histologic assessments of injury severity, body mass change, and circulating hormone concentrations were reported in our companion paper and are briefly described here to allow characterization of our model [2]. The average injury force and velocity ranged from 256–264 kilodyne and 120–122 mm/s, respectively, with no differences among groups. A near-complete loss of hindlimb locomotor function occurred in all SCI animals immediately after injury, with a gradual (expected) improvement in locomotor function occurring from days 7–21 post-injury. At sacrifice, hindlimb locomotor function remained below the threshold for voluntary weight-supported stepping, with no differences across SCI groups. Qualitative histological evaluation of the spinal cord at the injury epicenter verified the presence of a moderate/severe SCI in all groups, which was characterized by a thin layer of white matter in the ventral half of the cord with some myelin, axon, and collagen preservation and significant axonal loss. SCI animals exhibited an expected 10–15% reduction in body mass, which was not altered by TE or Scl-Ab. Serum sclerostin was similar in SHAM, SCI, and SCI+TE animals (group ranges 340 to 427 pg/ml) and could not be accurately measured in SCI+Scl-Ab animals because Scl-Ab exhibited cross-reactivity with the assay. Serum testosterone was ~50% lower in SCI versus SHAM, while SCI+TE exhibited a peak testosterone that was 70% higher than SHAM.

### Tissue mass

**Fig 1:**
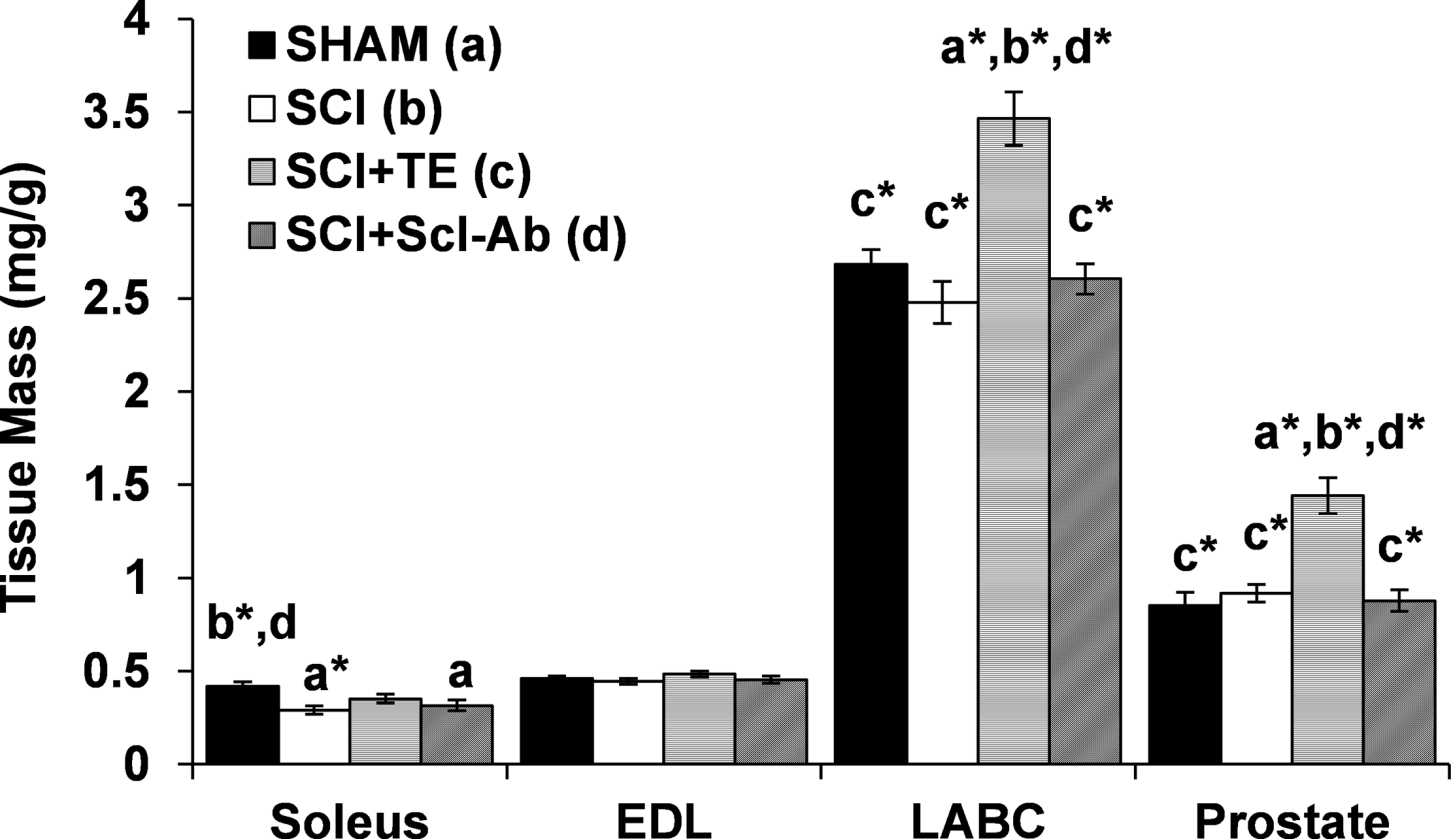
Muscle and prostate mass after sham surgery (T_8_ laminectomy) or moderate-severe spinal cord injury (SCI) alone or in combination with testosterone-enanthate (SCI+TE) or sclerostin-antibody (SCI+Scl-Ab). Values are means ± SEM, n = 9-10/group. Letters a-d indicate differences from respectively labeled groups at p<0.05 or *p<0.01 (a = vs SHAM; b = vs SCI; c = vs SCI+TE; d = vs SCI+Scl-Ab).

Tissue weights were corrected for body mass because of the expected body mass loss in all SCI groups. At sacrifice, soleus mass was 25–31% lower in SCI (p < 0.01) and SCI+Scl-Ab groups (p < 0.05) versus SHAM (Fig 1). In contrast, no difference in soleus mass was present among SCI+TE and SHAM animals. LABC mass and prostate mass were not different in SHAM, SCI, or SCI+Scl-Ab groups. In comparison, SCI+TE animals exhibited a 30–40% higher LABC mass (p < 0.001) and 57–70% higher prostate mass (p < 0.001) versus all other groups. No differences in EDL mass were present among groups. Among SCI groups, soleus mass was positively correlated with BBB score (r = 0.746, p <0.001), but not with serum testosterone. In contrast, LABC and prostate mass were positively correlated with serum testosterone (r = 0.679–0.703, p < 0.001), but not BBB score.

### Soleus fiber type distribution

**Fig 2:**
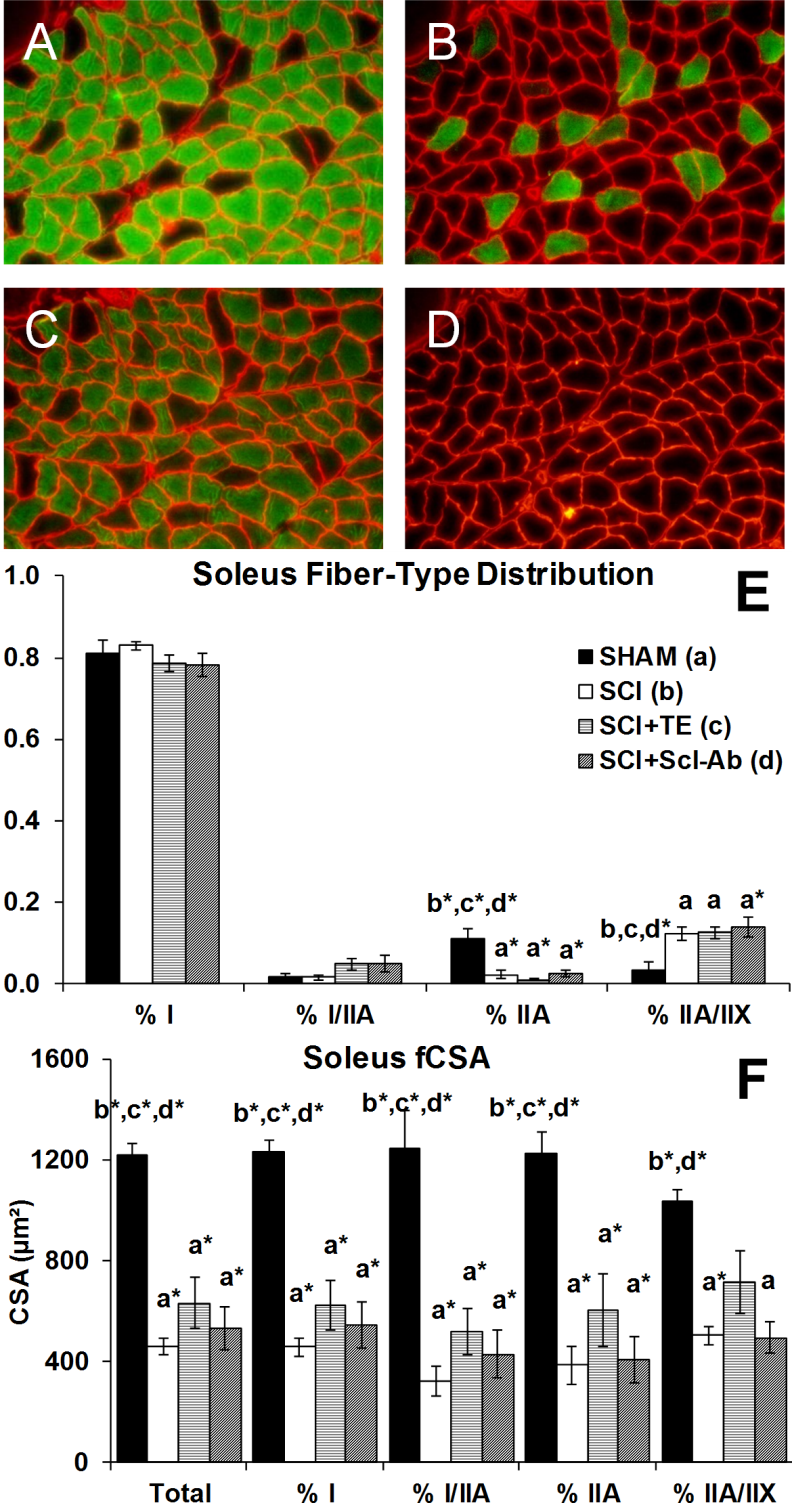
A-F. Soleus muscle fiber-type distribution and fiber cross-sectional area (fCSA) after sham surgery (T_8_ laminectomy) or moderate-severe spinal cord injury (SCI) alone or in combination with testosterone-enanthate (SCI+TE) or sclerostin-antibody (SCI+Scl-Ab). Panel A-D are representative images of serial cross-sections from soleus stained with monoclonal antibodies directed against (A) MHC-1 (stained green), (B) MHC-IIA (stained green), (C) MHC-IIX (unstained black, with all other MHC isoforms green), (D) MHC-IIB (green, none present). Panels E-F values are means ± SEM, n = 6-8/group. Letters a-d indicate differences from respectively labeled groups at p<0.05 or *p<0.01 (a = vs SHAM; b = vs SCI; c = vs SCI+TE; d = vs SCI+Scl-Ab).

**Fig 3:**
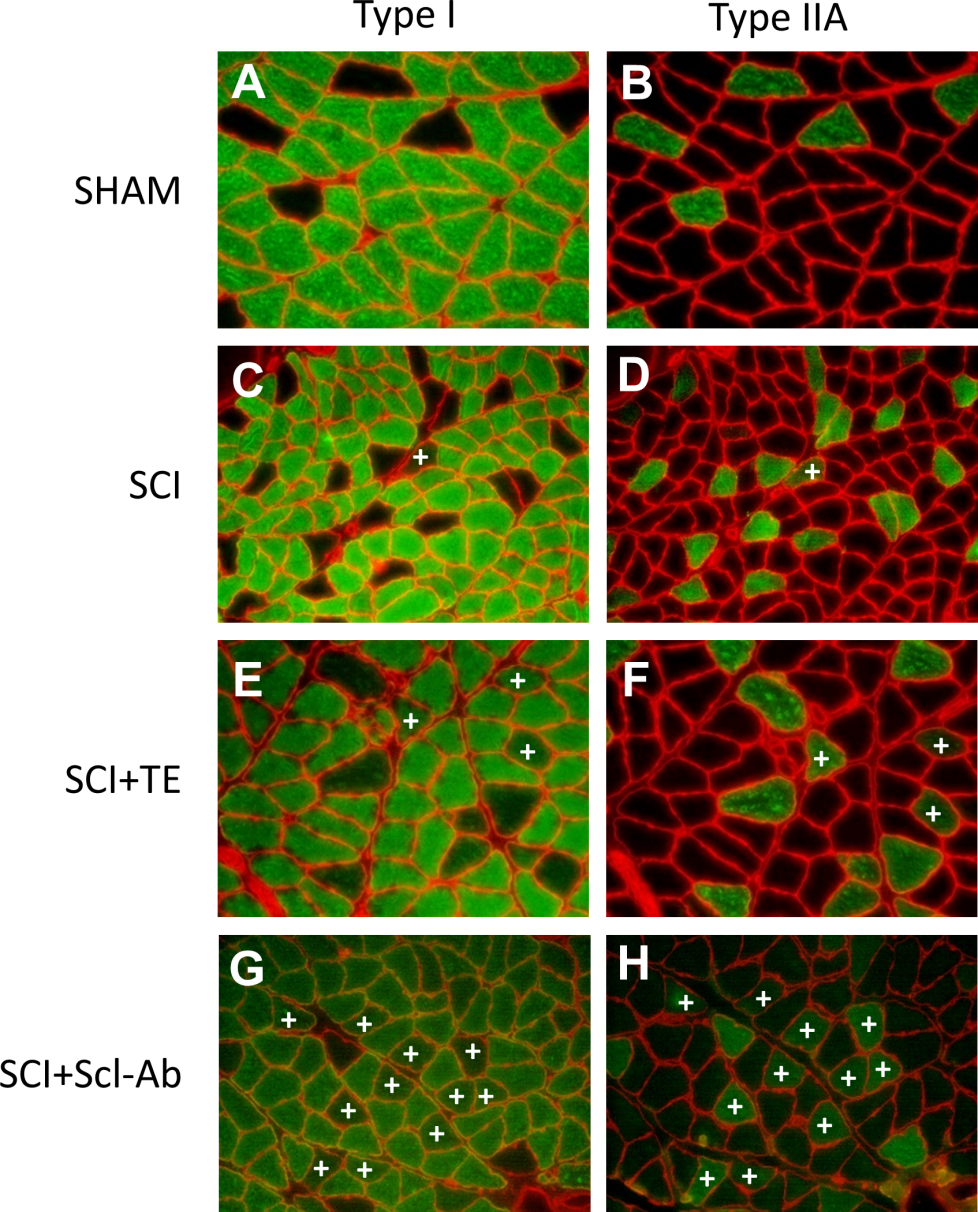
A-H. Representative histologic images of hybrid type I/IIA fibers after sham surgery (T_8_ laminectomy) or moderate-severe spinal cord injury (SCI) alone or in combination with testosterone-enanthate (SCI+TE) or sclerostin-antibody (SCI+Scl-Ab). Panels in left and right columns are representative serial cross-sections from soleus stained with monoclonal antibodies directed against MHC-1 (stained green) or MHC-IIA (stained green), respectively, for each group. +indicates hybrid I/IIA fibers. All images acquired at 20X magnification.

**Fig 4:**
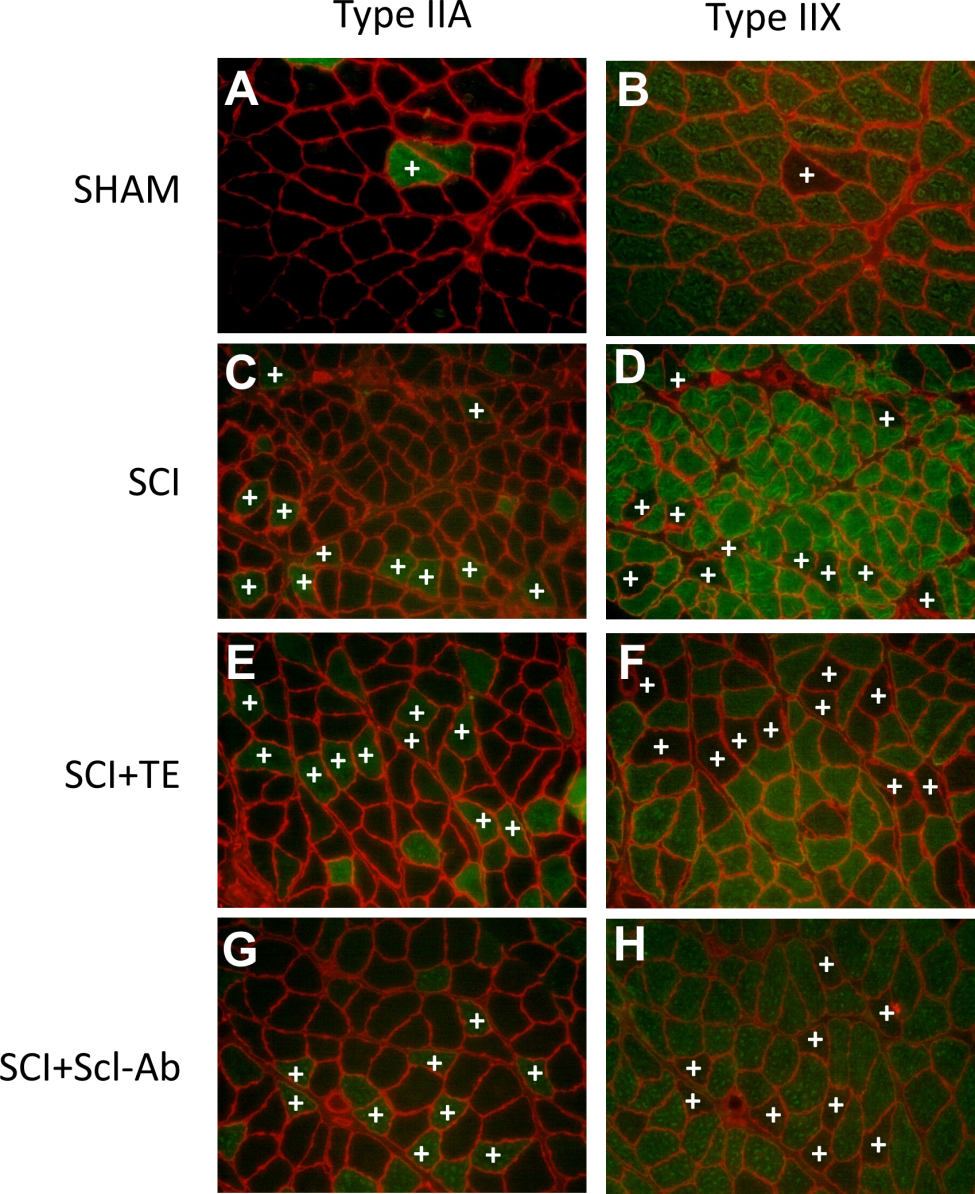
A-H. Representative histologic images of hybrid type IIA/IIX fibers after sham surgery (T_8_ laminectomy) or moderate-severe spinal cord injury (SCI) alone or in combination with testosterone-enanthate (SCI+TE) or sclerostin-antibody (SCI+Scl-Ab). Panels in left and right columns are representative serial cross-sections from soleus stained with monoclonal antibodies directed against MHC-IIA (stained green) or MHC-IIX (unstained), respectively, for each group. +indicates hybrid IIA/IIX fibers. All images acquired at 20X magnification.

Representative histologic staining for MHC-1, MHC-IIA, MHC-IIX, and MHC-IIB is presented in Fig 2A–2D. The fraction of type I fibers in soleus muscle ranged from 78–83% in all groups and was surprisingly unchanged by SCI, within the time frame of our study (Fig 2E). SHAM soleus displayed 11% type IIA fibers, while all SCI groups exhibiting a lower proportion of type IIA fibers vs SHAM (all p<0.001). Interestingly, we also observed a proportion of hybrid fibers that stained for both type I and IIA (Fig 3A–3H) or for both type IIA and IIX (Fig 4A–4H). The proportion of hybrid I/IIA fibers was ~2% in SHAM and no statistical differences were present across groups, although, the proportion of hybrid I/IIA fibers in SCI+TE and SCI+Scl-Ab groups was double that of other groups (non-significant). SHAM animals also exhibited ~3% IIA/IIX hybrid fibers, while 12–14% of all fibers displayed hybrid IIA/IIX characteristics in SCI (p < 0.05 vs SHAM), SCI+TE (p < 0.05 vs SHAM), and SCI+Scl-Ab (p < 0.01 vs SHAM). The number of IIX fibers was insufficient for analysis in all groups and no IIB fibers were observed (S1 File). Among SCI groups, serum testosterone was not associated with soleus fiber-type distribution. However, BBB score was positively associated with the proportion of hybrid I/IIA fibers (r = 0.647, p = 0.002).

### Soleus muscle fiber cross-sectional area

**Fig 5:**
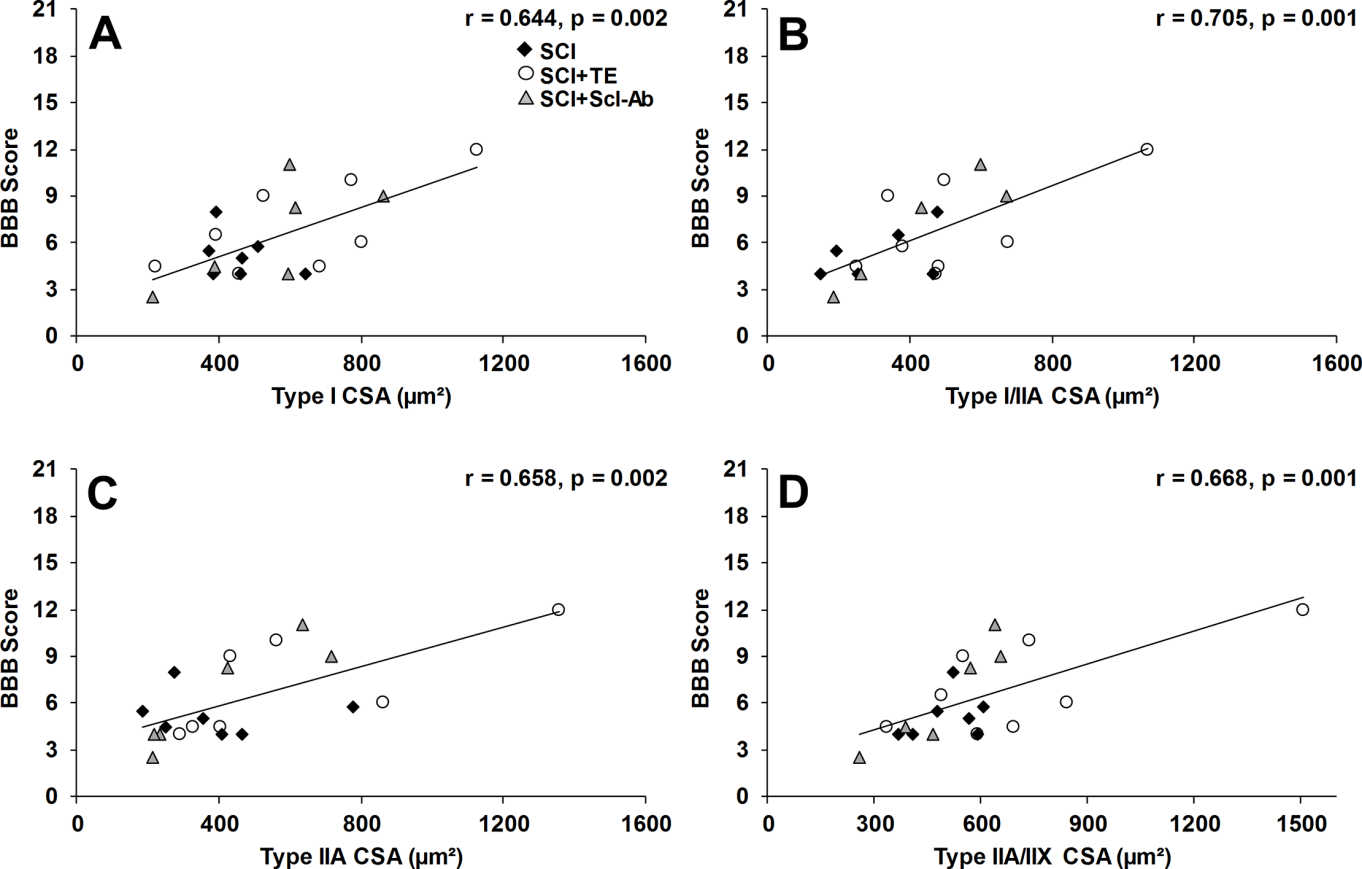
A-D. Pearson correlation coefficients for BBB score and muscle fiber cross-sectional area (fCSA) for animals that received spinal cord injury (SCI) alone or in combination with testosterone-enanthate (SCI+TE) or sclerostin antibody (SCI+Scl-Ab). Values represent individual animals, n = 20-21/analysis.

Soleus fCSA (inclusive of all fiber types) was 62% lower in SCI animals versus SHAM (p < 0.001, Figs 2F, 3A–3H and 4A–4H), characterized by 63% lower type I fCSA (p < 0.001), 74% lower type I/IIA hybrid fCSA (p < 0.001), 68% lower type IIA fCSA (p < 0.001), and 51% lower type IIA/IIX hybrid fCSA (p < 0.01). Scl-Ab did not prevent the reductions in total or fiber-type specific fCSA, with all values remaining similar to SCI. In comparison, TE partially prevented the reduction in hybrid IIA/IIX fCSA, exemplified by values that were 42–45% higher than SCI and SCI+Scl-Ab (non-significant) and not different than SHAM. Among all SCI groups, BBB score was highly, positively associated with total soleus fCSA (r = 0.665, p = 0.001) and all fiber types examined (Fig 5A–5D), while no significant associations were present among serum testosterone and soleus fCSA. When SCI groups were analyzed separately, no significant associations among BBB and any measure of soleus fCSA were present for untreated SCI animals. In comparison, several strong trends and significant associations were observed among BBB and measures of soleus fCSA for SCI+TE and SCI+Scl-Ab animals, respectively (Table 1).

**Table 1:** Pearson correlation coefficients for BBB score and muscle fiber cross-sectional area (fCSA) within groups of animals receiving spinal cord injury (SCI) alone or in combination with testosterone-enanthate (SCI+TE) or sclerostin antibody (SCI+Scl-Ab).

Group	N/group	Total fCSA	Type I fCSA	Type I/IIA fCSA	Type IIA fCSA	Type IIA/IIX fCSA
**SCI**	7	r = -0.262 p = 0.571	r = -0.382 p = 0.398	r = 0.475 p = 0.341	r = -0.053 p = 0.909	r = 0.326 p = 0.475
**SCI+TE**	8	r = 0.703 p = 0.052	r = 0.693 p = 0.057	r = 0.600 p = 0.115	r = 0.709 p = 0.074	r = 0.692 p = 0.057
**SCI+Scl-Ab**	6	r = 0.763 p = 0.078	r = 0.722 p = 0.105	r = 0.928 p = 0.023	r = 0.914 p = 0.011**†**	r = 0.931 p = 0.007**†**

Values are Pearson’s correlation coefficients, with respective p-values.

**†**indicates a statistically significant finding using the Holm-Bonferroni correction to control for potential type I error.

### Gene expression

**Fig 6:**
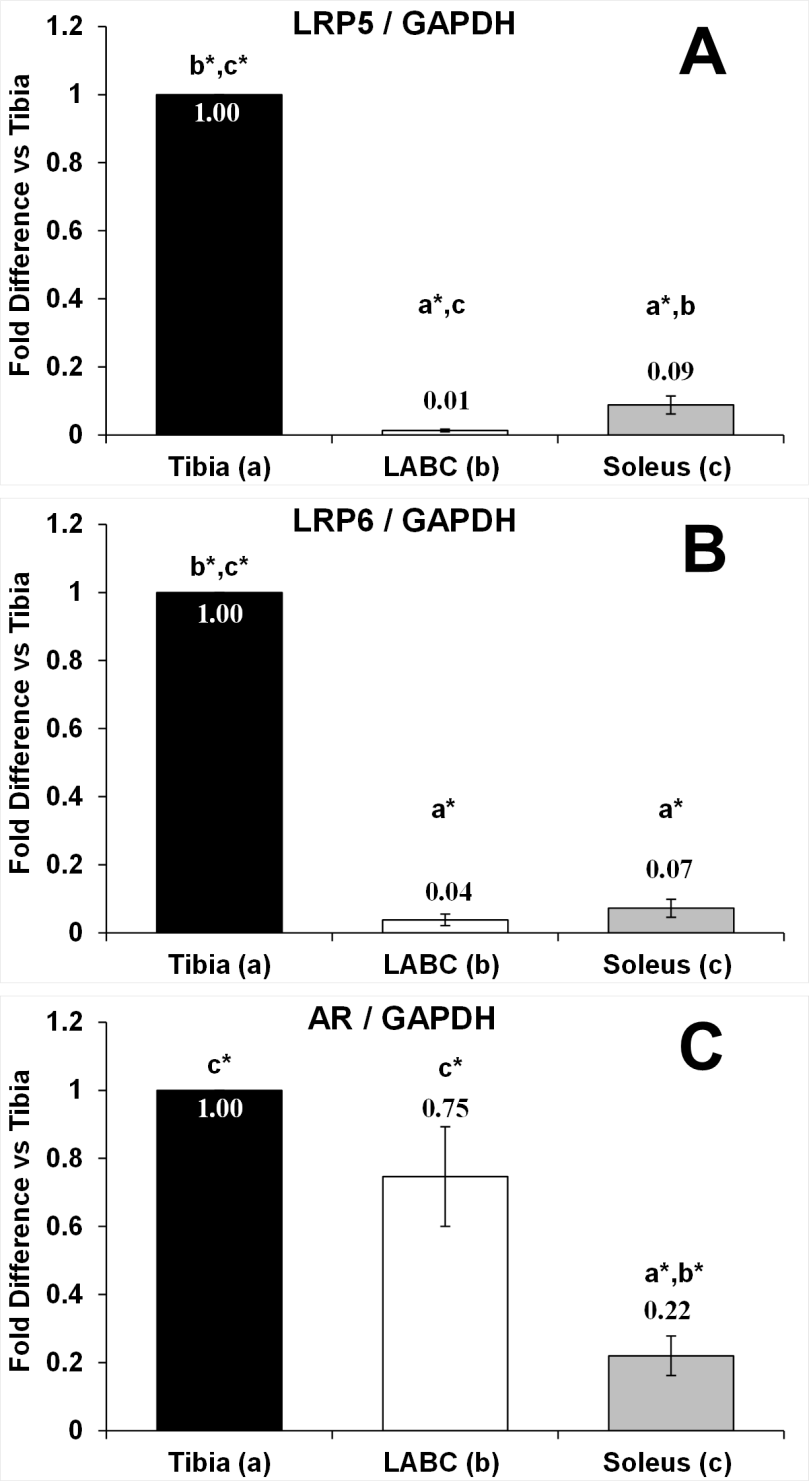
A-C. LDL receptor related protein 5 (LRP5) and 6 (LRP6), and androgen receptor (AR) gene expressions in tibia, levator ani/bulbocavernosus (LABC), and soleus from animals receiving sham surgery (T_8_ laminectomy). Values are means ± SEM [corrected for glyceraldehyde-3-phosphate dehydrogenase (GAPDH) as the housekeeping gene] and expressed relative to the tibia, n = 6/tissue. Letters a-c indicate differences from respectively labeled groups at p<0.05 or *p<0.01 (a = vs Tibia; b = vs LABC; c = vs Soleus).

LRP5, LRP6, and AR gene expressions were evaluated in tibia, soleus, and LABC from SHAMs. LRP5 and LRP6 expressions were 91–99% lower in soleus and LABC muscles in comparison to tibia (p < 0.001 for both, Fig 6A and 6B). AR expression was similar in the tibia and LABC, while being 70–78% lower in soleus (p < 0.001 vs tibia; p < 0.01 vs LABC, Fig 6C).

## Discussion

Pharmacologic strategies targeting preservation of musculoskeletal integrity have the potential to improve physical rehabilitation efforts after SCI. The principal findings of this investigation were that Scl-Ab did not prevent soleus atrophy after SCI and did not alter mass of the sublesional non-weight-bearing LABC muscle complex. We have previously reported that Scl-Ab and TE both prevented SCI-induced cancellous bone loss in rodents, albeit via differing cellular mechanisms [2]. Specifically, Scl-Ab increased osteoblast surface and cancellous bone formation, indicating direct bone anabolic effects, whereas TE reduced osteoclast surface with minimal effect on bone formation, indicating antiresorptive actions [2]. Herein, we examined the effects of Scl-Ab and TE on muscle preservation in the same animals from our previous report. The inability of Scl-Ab to induce myotrophic actions likely occurred because (1) the muscles we examined exhibited >90% lower mRNA expression of the sclerostin co-receptors (LRP5/LRP6) in comparison to the tibia, a tissue in which Scl-Ab produced potent bone anabolic actions after SCI [2,10] and/or (2) sclerostin was not present in a sufficient concentration within the circulation to attenuate LRP5/6-mediated Wnt-signaling *in vivo*. In this regard, the Wnt/β-catenin signaling pathway is anabolic in skeletal muscle [32] and sclerostin co-incubation has been shown to abolish Wnt3a-mediated C2C12 differentiation in culture, demonstrating that sclerostin antagonizes Wnt-signaling in an isolated murine muscle cell line [13]. However, LRP6 expression is fiber-type specific, with normal and atrophic human skeletal muscle expressing LRP6 only in type II fibers and C2C12 cells expressing LRP6 only in the presence of fast myosin [12], explaining the relatively low LRP6 mRNA expression that we observed in the soleus, a muscle comprised predominantly of type I fibers. In comparison, Tran et al reported >50% of cultured myoblasts from murine muscle were LRP5 positive, although, satellite cells for this experiment were isolated from the tibialis anterior [32], which is composed of predominantly type II fibers. Additionally, it is important to note that the sclerostin dose used by Huang et al [13] to abolish Wnt3a-signaling (100 ng/ml) was 200–300 times greater than the circulating concentrations present in our rodents, suggesting that supraphysiologic sclerostin concentrations may be necessary to inhibit LRP5/6-mediated Wnt-signaling in skeletal muscle, although, direct dose-response studies are needed to verify this contention. Interestingly, Battaglino et al reported that circulating sclerostin was elevated in individuals acutely after SCI, when bone loss is most severe [8], and Qin et al reported that mice with sclerostin gene deletion do not exhibit bone loss after SCI [9], indicating that elevated sclerostin influences bone loss after SCI. Others have also reported that Scl-Ab treatment completely prevented sublesional bone loss in the relative absence of mechanical loading [10,33,34,35], demonstrating the ability of Scl-Ab to protect against disuse-induced bone loss. Our findings complement these reports by providing the first evaluation of muscle morphology after Scl-Ab treatment, which supplies another layer of support indicating that the bone anabolic actions of Scl-Ab occur relatively independently of muscle strain, given that that no discernable differences in muscle fCSA or voluntary hindlimb locomotor recovery (BBB scores) were present among SCI and SCI+Scl-Ab groups. In contrast, the presence of musculoskeletal strain may be necessary for Scl-Ab to potentiate myotrophic responses, as has been reported with several other pharmacologic agents that produce anabolic responses only in loaded muscle [36,37]. Preliminary support for this contention is derived from our findings that indicate highly positive associations exist among BBB score and soleus type IIA fCSA (r = 0.914) and IIA/IIX fCSA (r = 0.931) in SCI+Scl-Ab animals, suggesting that the presence of mechanical strain influences the ability of sclerostin inhibition to promote myotrophic responses in type II fibers. As such, future research evaluating the anabolic potential of Scl-Ab in muscles with relatively high type II MHC expression remains warranted, especially when administered in combination with supplemental loading.

A secondary finding of our study is that TE increased mass of the LABC muscle complex after SCI, which supports previous findings from our laboratory [15,16], while producing less robust effects on soleus mass and fCSA in 5-month-old rats. In this regard, SCI resulted in a reduction in soleus mass and muscle fCSA across all fiber types, along with an expected slow-to-fast fiber type transition that was best exemplified by a higher proportion of hybrid type IIA/IIX fibers in SCI animals versus SHAM. Similarly, Talmadge et al reported that spinal cord transection resulted in increased soleus type IIA/IIX hybrid fibers within 15 days of injury [38], and that the slow-to-fast fiber transition in the soleus continually developed for upwards of 1-year after SCI, at which time the soleus exhibited nearly 50% type IIA/IIX hybrid fibers [39]. In our current study, soleus mass and fCSA were not different among SCI vs SCI+TE, although, the mean values for these muscular outcomes were directionally higher by ~35–50% in animals receiving TE. Interestingly, we have previously reported that TE fully preserved soleus fCSA [27] and increased LABC fCSA in ambulatory rats following orchiectomy [28]. In contrast, TE only partially prevented soleus atrophy in young (3-month-old) male rats after moderate-severe contusion SCI in our previous study [16]. The differing musculoskeletal responses among fully ambulatory orchiectomized animals and the SCI animals in our current and previous reports appears to indicate that mechanical strain influences the myotrophic effects of TE, as has been reported in humans [40]. Indeed, we have previously observed that young male SCI rodents exhibit a dose-dependent improvement in voluntary hindlimb locomotor activity in response to TE, which was accompanied by increasing preservation of soleus mass [16]. Herein, no discernable differences in BBB scores were present among SCI groups. However, soleus mass and fCSA were highly positively correlated with BBB scores across all SCI groups, which supports the contention that mechanical strain influences rodent soleus mass after SCI. In addition, we observed several strong, albeit non-significant, trends among BBB and soleus fCSA measures in SCI+TE animals, suggesting that differences in locomotor recovery may influence recovery of muscle fCSA after SCI animals receiving TE. Indeed, Byers et al also reported that testosterone profoundly improved muscle fCSA in the vastus lateralis of young rodents after moderate contusion SCI, a model that exhibits spontaneous voluntary locomotor recovery after injury [21]. In comparison, Wu et al reported that testosterone treatment alone did not ameliorate muscle loss in several plantar flexor muscles in young rodents after spinal cord transection, a model that exhibits negligible sublesional weight bearing after injury, but that testosterone completely prevented the supplemental muscle loss induced by methylprednisolone [23,24]. Although, at least one group has reported preservation of plantar flexor and vastus lateralis muscle CSA in rodents after spinal cord transection [22].

An alternative explanation for the differing myotrophic responses among various rodent muscles after androgen treatment is that AR expression differs among muscles. For example, TE profoundly increased mass of the non-weight bearing LABC muscle, but produced minimal effects on soleus fCSA, likely because AR expression in LABC was >3-fold higher than soleus. Similarly, TE did not alter EDL mass in this study, likely because the rodent EDL is considered to be relatively androgen insensitive due to a low AR expression [4]. Interestingly, the tibia and LABC muscle from our animals exhibited roughly similar AR expressions and both tissues responded positively to TE treatment after SCI, supporting the contention that AR expression influences tissue androgen responsiveness. The aforementioned findings highlight the need to carefully evaluate tissue-specific AR expression when assessing androgen responsiveness in model species, especially given the plethora of data supporting the notion that AR expression regulates androgen responsiveness across species [4]. This notion is also important because several small clinical studies have reported that testosterone replacement therapy (TRT) increases lean mass and energy expenditure [18,19] and muscle cross-sectional area in the paralyzed limbs of men with motor-complete SCI [20], while the findings from animal models are somewhat contradictory.

Several limitations of this study also merit mention. First, we expected to observe a lower proportion of type I fibers and higher proportions of type I/IIA (hybrid) fibers in soleus of SCI animals, as has been reported by several other groups within a similar time frame [39,41]. However, the distributions of these fiber types did not differ among SHAM or SCI animals in our study, despite a >50% lower fCSA after SCI. The most likely explanation for this observation is that we utilized the IH impactor to induce a moderate-severe (250 kdyne) contusion SCI, which is characteristically different than the New York University (NYU) weight-drop system utilized by Stevens et al [41] and the complete spinal cord transection model utilized by Talmadge et al [38,39], especially in terms of injury severity. Indeed, the force produced with the 25-mm (10-g) NYU weight-drop system compares more directly with a 400-kdyne IH impactor injury [42], indicating that our 250-kdyne (IH impactor) SCI model is somewhat less severe than that of Stevens et al, thus, less likely to exhibit the dramatic slow-to-fast fiber transition observed in more severe SCI models [39,41]. Indeed, Hutchinson et al reported that animals receiving a contusion SCI, that is more comparable to our model, exhibited ~20% lower soleus mass than controls and no difference in soleus type I MHC expression at 1-week, 2-weeks, or 10-weeks post-injury [43], which appears consistent with our findings. Alternatively, we also observed a relatively lower proportion of soleus type I fibers in SHAM animals (~80%) in comparison to the 85–90% type I fibers observed in previous studies [39,41], which may have underestimated the effect of SCI on type I fiber distribution. Regardless, we are confident in our data because (1) we evaluated a total of 300–350 muscle fibers from each animal, exceeding that reported by others [41,44], (2) SCI produced the expected reduction in muscle fCSA across all fiber types, and (3) we observed a higher proportion of type IIA/IIX fibers in all SCI groups, indicative of the expected slow-to-fast fiber type transition in soleus [38,39]. Second, we were unable to demonstrate increased circulating sclerostin in our rodent model acutely after SCI, which is in contrast to what has been reported in humans that are undergoing rapid bone loss subsequent to SCI [8]. The most likely explanation for this is that we evaluated circulating sclerostin at day 21, after the majority of cancellous bone loss and accompanying osteocyte loss occurs in response to contusion SCI [45], which is important given that sclerostin is predominantly osteocyte-derived. Regardless, sclerostin was present in the circulation of SHAM and SCI animals, albeit in relatively low concentrations, and our published data indicates that systemic Scl-Ab treatment potently stimulated bone formation and completely prevented SCI-induced bone loss in these animals [2], demonstrating effectiveness of the drug dose we selected.

## Conclusions

In summary, Scl-Ab produced no discernable benefit or detriment to soleus muscle morphology or fiber type distribution after moderate-severe contusion SCI, likely because (1) the sclerostin LRP5/LRP6 co-receptors are only minimally expressed in the rat soleus, which is composed predominantly of type I fibers, (2) circulating sclerostin may not have been present in sufficient concentration to attenuate LRP5/6-mediated Wnt-signaling *in vivo*, and/or (3) mechanical stimuli may be necessary for Scl-Ab to promote anabolic response in skeletal muscle, as has been reported with several other myotrophic agents [36,37]. In contrast, TE completely preserved mass of the non-weight-bearing LABC muscle complex, but only partially prevented soleus atrophy, with the differences among muscles likely being explained by the relatively higher AR expression in LABC. These results highlight the importance of selecting appropriate muscles and examining hormone receptor expression in target tissues of preclinical model species when initiating pharmacologic therapy intended to promote myotrophic effects.

## Supporting information

S1 File Soleus fiber-type counts and fiber cross-sectional area (fCSA). Values represent individual fCSA for each fiber evaluated, with fiber-type delineated, from all animals included in the study.
(XLSX)
